# A sensory neuroprosthesis enhances recovery from treadmill-induced stumbles for individuals with lower limb loss

**DOI:** 10.1038/s41598-025-85788-4

**Published:** 2025-01-11

**Authors:** Suzhou Li, Ronald J. Triolo, Hamid Charkhkar

**Affiliations:** 1 Biomedical Engineering, Case Western Reserve University, Cleveland, OH 44106 USA; 2 Louis Stokes Cleveland Veteran Affairs Medical Center, Cleveland, OH 44106 USA

**Keywords:** Musculoskeletal, Stumbles, Trip recovery, Neuroprosthesis, Sensorimotor control

## Abstract

Over 50% of individuals with lower limb loss report a fear of falling and avoiding daily activities partly due to a lack of plantar sensation. Providing direct somatosensory feedback via neural stimulation holds promise for addressing this issue. In this study, three individuals with lower limb loss received a sensory neuroprosthesis (SNP) that provided plantar somatosensory feedback corresponding to prosthesis-floor interactions perceived as arising from the missing foot generated by electrically activating the peripheral nerves in the residuum. Participants walked on a treadmill while receiving perturbations involving brief increases in the belt speed. Perturbations were initiated during early stance and randomly delivered to intact and prosthetic sides with the SNP active or inactive. With the SNP active, participants exhibited decreased trunk angular sway and peak trunk flexion angular velocity during recovery from both prosthetic and intact side perturbations. For prosthetic side perturbations, peak ground reaction force magnitudes decreased when the SNP was active. For intact side perturbations, peak ground reaction force magnitudes increased on the prosthetic side’s first recovery step after the perturbation, which resulted in a more symmetric recovery because the force approached the response on the intact side’s first recovery step following a prosthetic side perturbation. These results suggest participants integrated the feedback from the SNP into their sensorimotor control for maintaining stability and gained confidence in relying on their prosthetic limb during recovery. Restoring plantar sensation with a SNP for individuals with lower limb loss could lead to reduced risk of falling by improving recovery from trips.

**Supplementary Information:**

The online version contains supplementary material available at 10.1038/s41598-025-85788-4.

## Introduction

There are currently more than a million individuals living with lower limb loss (LLL) in the United States^1^. Over half of these individuals report falling at least once within a year, with a similar percentage experiencing a fear of falling, leading many to avoid daily activities and adopt a sedentary lifestyle^2^. Lower limb prosthesis users identify walking with stability and maintaining balance as key functional needs for their prosthetic devices, which are closely interconnected with the desires to improve their perception of safety, increase confidence in using their prosthesis, and participate more fully in their communities and social lives^3^.

One critical component of balance is plantar sensation. This sensation provides the body with feedback about foot-floor contact, informing the sensorimotor control system to generate appropriate motor commands for maintaining stability^4,5^. Previous studies on balance have highlighted the importance of plantar sensation and its contribution to the control of weight transfer during recovery steps from forward falls^5–7^. Based on these findings, orthotic solutions such as raised-edge insoles have been developed to reduce the risk of falls in the elderly by enhancing feedback about the magnitude and distribution of loads applied to the plantar surface of their feet^6,7^. Similarly, a training program aimed at improving plantar surface sensitivity in the elderly has also been shown to improve stability and confidence in their ability to recover after a fall^8^.

Individuals with LLL, however, no longer have this critical plantar sensation. Instead, most must rely on limited feedback from interactions between their residual limbs and prostheses sockets. As such, when experiencing a stumble, they exhibit recovery strategies which are different than their able-bodied peers. They attempt to bring their intact feet back in contact with the floor as soon as possible, indicating they have more confidence in using their intact limbs than their prostheses due to the quality of the sensory information the intact limb provides^9^. The altered recovery strategies adopted by prosthesis users could introduce instabilities and even introduce hopping or skipping which bring both feet off the floor simultaneously making controlling the body’s mass and remaining upright even more challenging^9^. Current interventions for reducing the risk of falls after lower limb amputation focus on improving lower body strength and balance, which has been shown to improve confidence and mobility^10^. Furthermore, teaching specific motor skills to minimize an impact after a fall can mitigate potential injuries^11,12^. Fall arrest and floor-to-rise training, for instance, are employed by physical therapists during rehabilitation after amputation to address the reality that individuals with LLL will fall and to mitigate the physical and psychological toll of those falls^11^. However, such interventions are time and effort intensive and do not address the instability and compromised recovery due to missing plantar sensation.

**Fig. 1:**
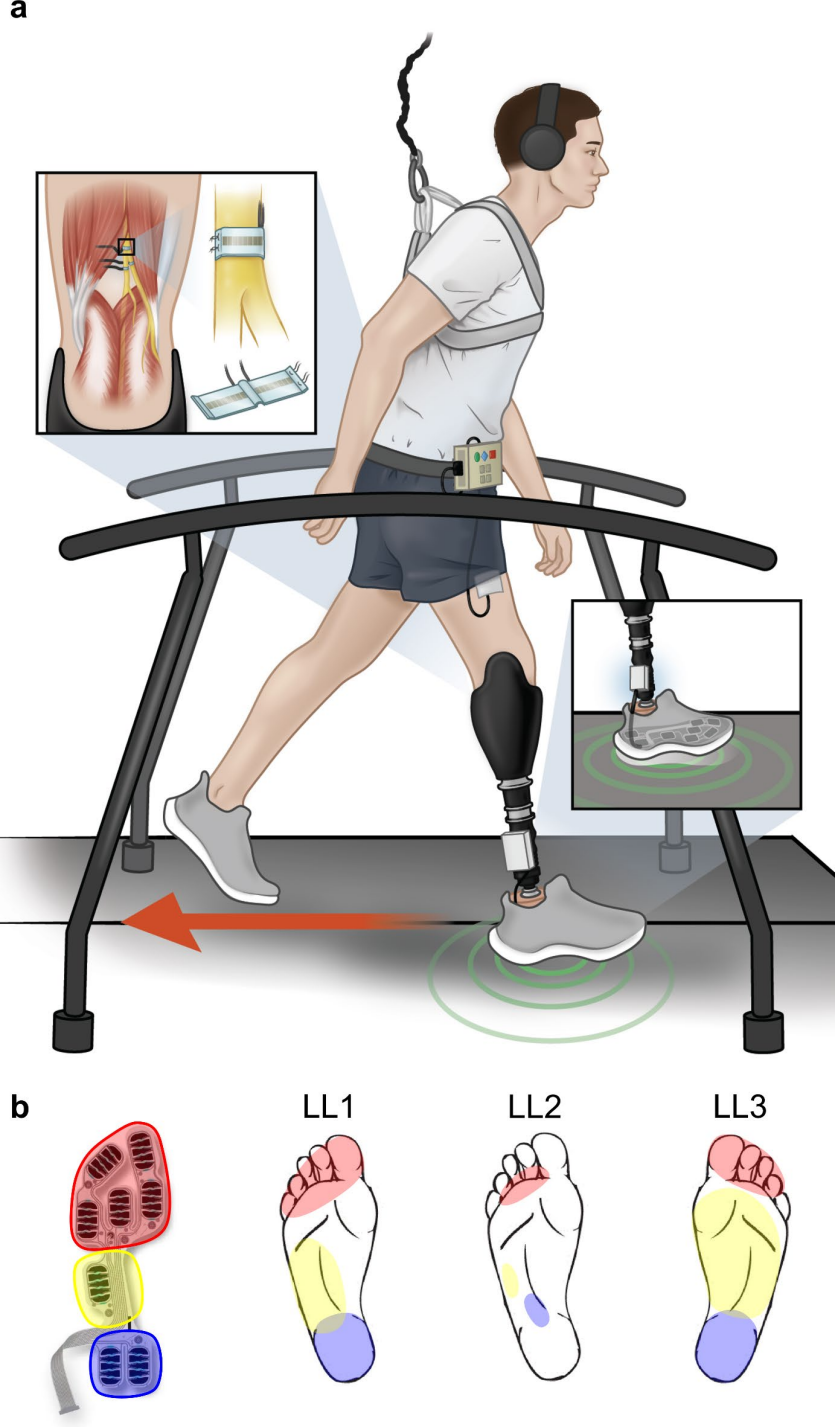
Sensory neuroprosthesis (SNP) technology and elicited sensations. (**a**) High-density multi-contact nerve cuff electrodes implanted around the remaining peripheral nerves in the participants’ residual limbs. Electrical stimulation through the electrode contacts elicits sensations perceived as originating from the missing foot. Sensation intensity and location are modulated by foot-floor interactions detected via a force sensing insole, restoring plantar somatosensory feedback during gait. (**b**) Participant-reported perceived locations of elicited sensations corresponding to pressure on the heel (blue), midfoot (yellow), and metatarsal (red) regions. The force-sensing insole is divided into three regions, where pressure applied to each region was determined by the average of the readings from sensor pads within each region.

Our team has developed a sensory neuroprosthesis (SNP) that restores somatosensation perceived as if arising from the missing feet of individuals with LLL. High-density multi-contact nerve cuff electrodes installed around the peripheral nerves remaining in the residual limb above the knee (i.e. the sciatic and tibial nerves) deliver small electric currents to activate the afferent fibers. This activation elicits sensations that correspond to the location and magnitude of loads applied to the prosthetic foot (Fig. 1a and b)^13^. We have shown that these electrically elicited sensations enhance standing stability, especially when sensory information from visual and vestibular inputs are compromised^14^; aid in navigating challenging terrains^15^; and improve perception of limb movement^16^. Furthermore, the sensation preserves the reflex circuitry in static postures mimicking gait, which suggests that it does not impede spinal reflex mechanisms that are important in maintaining stability^17^. Other groups using different neural interface technologies to activate afferent fibers have shown that the restored sensation results in more stable gait^18^ and reduced energy expenditure while walking^19^. While these reports provide some evidence on the functional benefits of restored plantar sensation, it has yet to be shown whether the electrically elicited plantar sensations from the SNP or other neural interface technologies can alter stumble recovery strategies and affect the ability of the user to compensate for destabilizing events, potentially reducing the risk of falls.

**Fig. 2:**
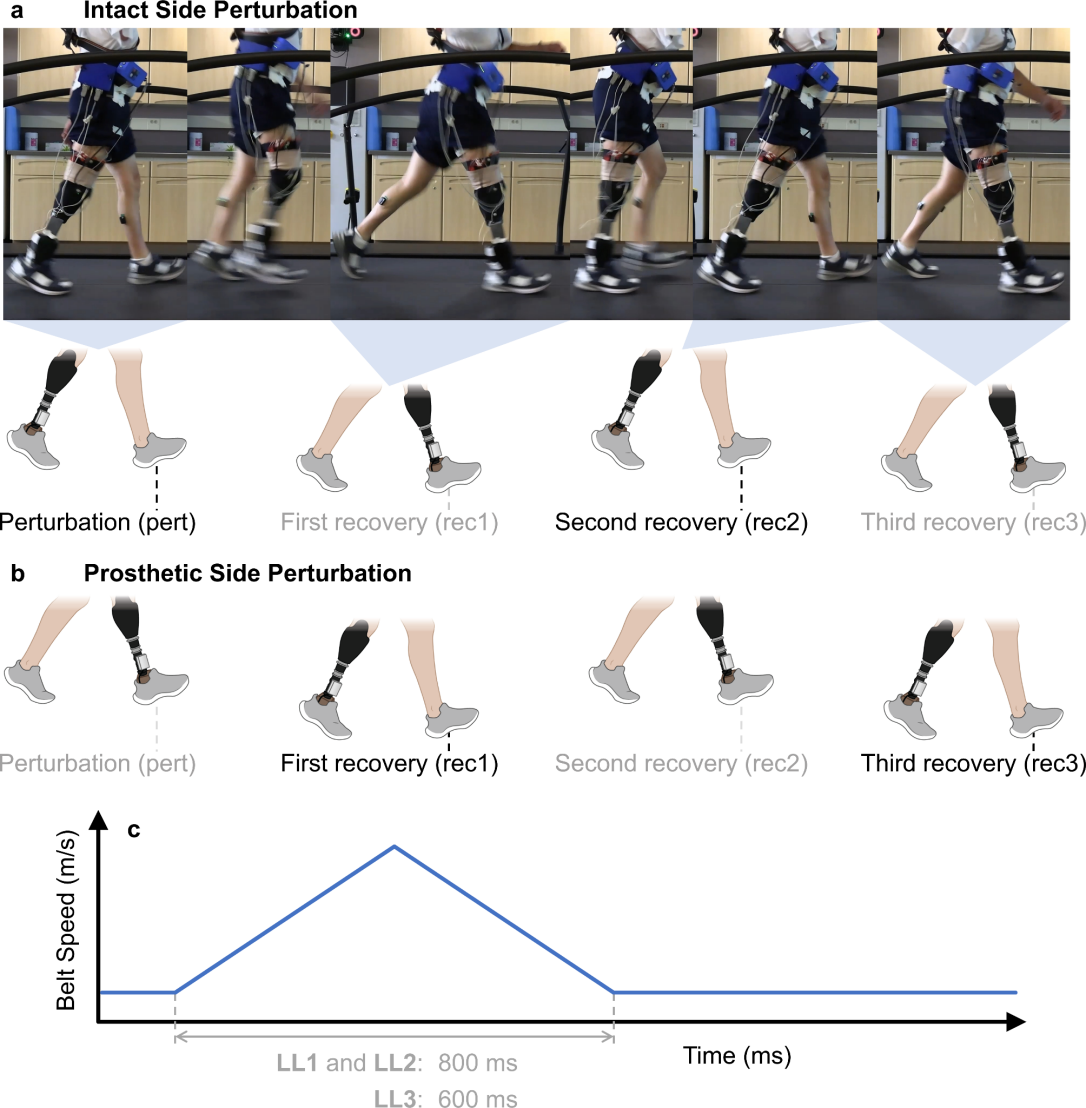
Treadmill-induced stumbles. (**a**) Intact side perturbation: treadmill belt accelerates during early stance of the intact limb. (**b**) Prosthetic side perturbation: stepping pattern following acceleration of the treadmill belt during prosthetic limb early stance. (**c**) Perturbations consist of the treadmill belt accelerating at 3 m/s^2^ for the first half and decelerating at the same rate for the second half. Data analysis encompasses the perturbation (pert) and three subsequent recovery steps (rec1, rec2, and rec3).

Trip recovery requires a quick and coordinated full-body response to counteract the forward rotation that can be induced by encountering an obstacle or by a controlled perturbation^20,21^. Trunk kinematics have been identified as important indicators of fall risk^21–23^. To investigate stumble recovery in a safe laboratory setting, treadmill-induced perturbations have been employed because they produce a forward rotation of the trunk similar to real-world trip recovery scenarios (as shown in Fig. 2a and b)^24^. These perturbations can serve dual purposes: as valuable research tools and as training methods to improve recovery skills, with retention observed for up to six months^25^. However, despite their utility in enhancing recovery responses, they still fall short of addressing the critical loss of sensory feedback from the missing foot of individuals with LLL and its contribution to stability and recovery.

Building upon these established methods, our study characterized stumble recovery in individuals with LLL who received the SNP. We focused on trunk kinematics and ground reaction forces (GRFs) during treadmill-induced perturbations (as shown in Fig. 2c) to characterize the recovery dynamics. Three individuals with unilateral transtibial or knee disarticulation amputations participated in the study. We hypothesized that with the SNP, participants would exhibit enhanced trunk control and limb loading profiles during recovery compared to conditions without the neural sensory feedback. We anticipated the enhanced trunk control would be evident by decreased trunk sway and reduced peak trunk flexion angular velocity, and improved limb loading would manifest as decreased GRF magnitudes on the recovery steps.

## Results

### Restoration of plantar sensation with SNP

All three participants, despite their different levels of amputation (LL1 and LL2 with trans-tibial amputation, LL3 with knee disarticulation amputation), reported discrete sensations from the heel, midfoot, and metatarsal regions of their missing foot when applying pressure to those corresponding areas of their prosthetic foot while the SNP was active. The locations of elicited percepts for each participant shown in Fig. 1b were identified through thresholding and mapping experiments described in previous work from our group^13–15^. The elicited sensations were described as comfortable, with modalities such as pressure, and similar in intensity to those felt in their intact foot.

### SNP decreased trunk kinematics during stumble recovery

**Fig. 3:**
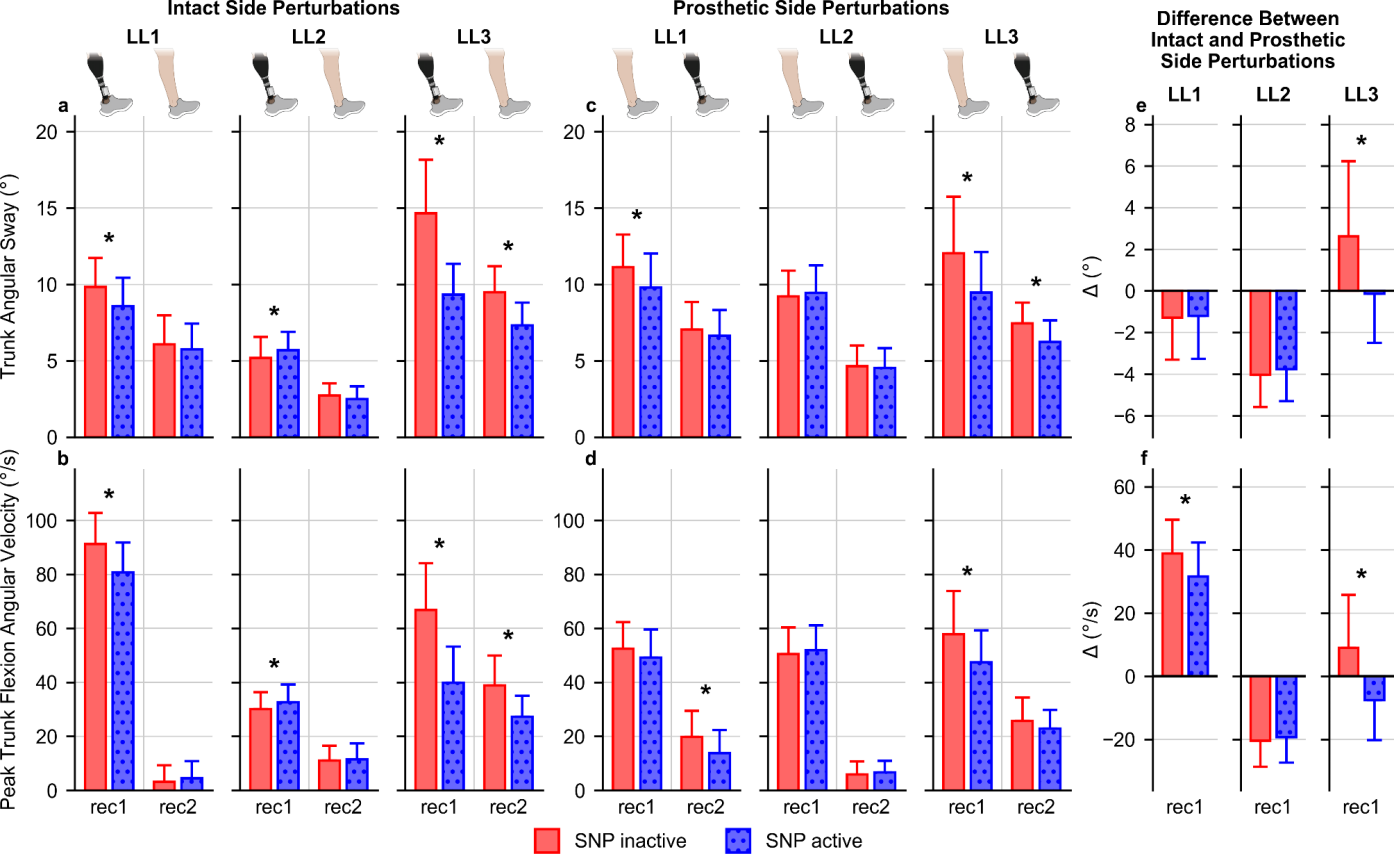
SNP effects on trunk kinematics during stumble recovery. (**a**) Trunk angular sway and (**b**) peak trunk flexion angular velocity for the first (rec1) and second (rec2) recovery steps after an intact side perturbation (SNP inactive: n = 58 for LL1, n = 62 for LL2, and n = 48 for LL3; SNP active: n = 59 for LL1, n = 64 for LL2, n = 53 for LL3). (**c**) Trunk angular sway and (**d**) peak trunk flexion angular velocity for the first and second recovery steps after a prosthetic side perturbation (SNP inactive: n = 62 for LL1, n = 66 for LL2, and n = 44 for LL3; SNP active: n = 62 for LL1, n = 65 for LL2, n = 52 for LL3). The leg (intact or prosthetic) initiating each recovery step (intact side perturbation: prosthetic for rec1 and intact for rec2, prosthetic side perturbation: intact for rec1 and prosthetic for rec2) can be seen overlaid on the top of (**a**) and (**c**). Differences between intact and prosthetic side perturbations for the mean (**e**) trunk angular sway and (**f**) peak trunk flexion angular velocity during the first recovery step. Positive differences indicate that the mean metric after intact side perturbations was greater than that after prosthetic side perturbations, and the converse is true for negative differences. Data for SNP inactive are represented in red, and data for SNP active are represented in blue. Each axis represents data from each individual participant. Bar heights represent the means, and the error bars represent the standard deviation (pooled standard deviation for **e** and **f**). * *P* < 0.05.

The SNP decreased trunk movement after intact and prosthetic side perturbations for LL1 and LL3. For intact side perturbations, significant interactions between recovery steps and SNP were observed for both trunk angular sway (LL1: F(3, 345) = 4.1, *P* = 0.012; LL3: F(3, 297) = 31.3, *P* < 0.001) and peak trunk flexion angular velocity (LL1: F(3, 345) = 17.3, *P* < 0.001; LL3: F(3, 297) = 32.2, *P* < 0.001). With SNP active and during the first recovery step, LL1 and LL3 exhibited reductions in trunk angular sway by 1.3 ± 0.3° (mean ± standard error) and 5.3 ± 0.6°, respectively (Fig. 3a, Table S1, both: *P* < 0.001), and in peak trunk flexion angular velocity by 10.5 ± 2.1°/s and 26.9 ± 3.1°/s, respectively (Fig. 3b, Table S2, both: *P* < 0.001). LL3 showed additional decreases in trunk sway by 2.2 ± 0.3° (*P* < 0.001) and 3.1 ± 0.5° (*P* < 0.001) during the second (Fig. 3a, Table S1) and third (Table S1) recovery steps respectively, and a decrease in peak trunk flexion angular velocity by 11.6 ± 1.9°/s during the second recovery step (*P* < 0.001, Fig. 3b, Table S2).

For prosthetic side perturbations, significant interactions between recovery steps and SNP activation were observed for trunk angular sway in both LL1 (F(3, 366) = 5.3, *P* = 0.002) and LL3 (F(3, 282) = 5.2, *P* = 0.012). LL3 also exhibited this interaction for peak trunk flexion angular velocity (F(3, 282) = 3.2, *P* = 0.041), while LL1 showed the main effects of SNP (F(1, 366) = 23.1, *P* < 0.001) and recovery steps (F(3, 366) = 513.9, *P* < 0.001) without interaction (F(3, 366) = 1.3, *P* = 0.280). When the SNP was active, LL1 and LL3 decreased their trunk angular sway during their first recovery step by 1.3 ± 0.4° and 2.6 ± 0.7°, respectively (Fig. 3c, Table S3, both: *P* < 0.001). LL3 also showed a reduction of 1.2 ± 0.3° during the second recovery step (Fig. 3c, Table S3, *P* < 0.001). For peak trunk flexion angular velocity, LL1 exhibited an overall average decrease of 3.6 ± 0.8°/s across recovery steps, with specific decreases of 3.3 ± 0.9°/s (*P* < 0.001) and 6.0 ± 1.6°/s (*P* < 0.001) on their perturbation (Table S4) and second (Fig. 3d, Table S4) recovery steps, respectively. LL3 exhibited decreases of 10.3 ± 2.8°/s (*P* < 0.001) and 4.7 ± 1.5°/s (*P* = 0.003) during their first (Fig. 3d, Table S4) and third (Table S4) recovery steps, respectively.

Unlike LL1 and LL3, LL2 showed only minimal SNP effects to recovery from intact side perturbations, where a significant interaction was observed between recovery steps and the SNP for both trunk angular sway (F(3, 372) = 3.8, *P* = 0.013) and peak trunk flexion angular velocity (F(3, 372) = 2.8, *P* = 0.049). LL2 exhibited a small but significant increase in trunk sway by 0.5 ± 0.2° (Fig. 3a, Table S1, *P* = 0.029) and peak trunk flexion angular velocity by 2.5 ± 1.1°/s (Fig. 3b, Table S2, *P* = 0.032) during the first recovery step when the SNP was active. LL2 demonstrated recovery responses to prosthetic side perturbations as shown by the significant effect of the recovery step on trunk sway (Fig. 3c, F(3, 387) = 873.9, *P* < 0.001) and peak trunk flexion angular velocities (Fig. 3d, F(3, 387) = 1200.8, *P* < 0.001). However, no significant SNP effects for trunk sway (F(1, 387) = 0.1, *P* = 0.731) and peak trunk flexion angular velocity (F(1, 387) = 0.1, *P* = 0.715) nor interaction effects for trunk sway (F(3, 387) = 0.6, *P* = 0.577) and peak trunk flexion angular velocity (F(3, 387) = 0.8, *P* = 0.438) were observed.

Analysis of the first recovery step revealed SNP-associated changes in the asymmetry between intact and prosthetic side perturbation responses. LL3 exhibited significant differences in asymmetry for both trunk angular sway (Fig. 3e, T(147.6) = 2.8, *P* = 0.007) and peak trunk flexion angular velocity (Fig. 3f, T(165.8) = 16.6, *P* < 0.001), and LL1 exhibited the difference only in peak trunk flexion angular velocity (Fig. 3f, T(230.5) = 7.3, *P* < 0.001) but not in trunk angular sway (Fig. 3e, T(234.3) = -0.09, *P* = 0.932). In contrast, LL2 did not experience significant changes in either trunk angular sway (Fig. 3e, T(235.4) = -0.3, *P* = 0.793) or peak trunk flexion angular velocity (Fig. 3f, T(227.5) = -1.1, *P* = 0.269).These changes indicated a trend where prosthetic side recovery kinematics more closely resembled those of intact side recovery when the SNP was active.

In summary, LL1 and LL3 demonstrated consistent reductions in both trunk angular sway and peak trunk flexion angular velocity with SNP active for both intact and prosthetic side perturbations. In contrast, LL2 showed slight increases in trunk angular sway and peak trunk flexion angular velocity, but only after intact side perturbations. In addition, the asymmetry in trunk kinematics between intact and prosthetic side perturbation responses reduced for LL1 and LL3.

### Ground reaction forces and confidence during stumble recovery

**Fig. 4:**
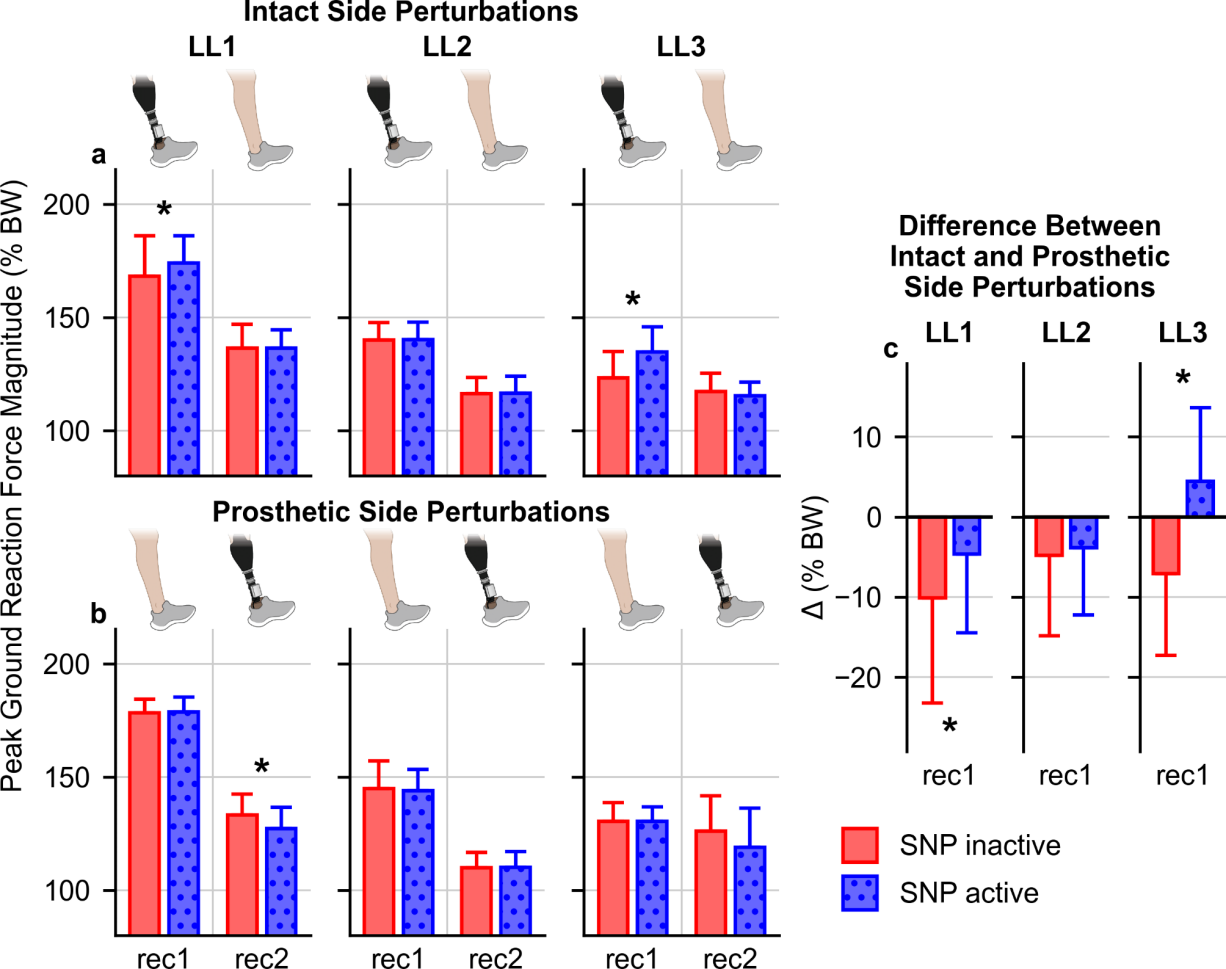
Perturbation side specific SNP effects on peak ||GRF||. (**a**) Peak ||GRF|| for the first (rec1) and second (rec2) recovery steps after intact side perturbations (SNP inactive: n = 54 for LL1, n = 57 for LL2, n = 44 for LL3; SNP active: n = 68 for LL1, n = 58 for LL2, n = 50 for LL3). (**b**) Peak ||GRF|| for the first and second recovery steps after prosthetic side perturbations (SNP inactive: n = 57 for LL1, n = 49 for LL2, n = 37 for LL3; SNP active: n = 61 for LL1, n = 49 for LL2, n = 42 for LL3). (**c**) Differences between intact and prosthetic side perturbations for the mean peak ||GRF|| during the first recovery step, where positive differences indicate that the mean intact side perturbation peak ||GRF|| was higher than that after prosthetic side perturbations. Red represents SNP inactive, and blue represents SNP active. Each axis represents data from each individual participant. Bar heights represent the means, and the error bars represent the standard deviation (pooled standard deviation for **c**). * *P* < 0.05.

Analysis of GRFs revealed distinct recovery patterns for intact and prosthetic side perturbations. During recovery from intact side perturbations, both LL1 and LL3 exhibited significant interactions between recovery steps and SNP on peak body weight normalized GRF magnitude, ||GRF|| (F(3, 360) = 3.2, LL1: *P* = 0.048; LL3: F(3, 276) = 12.2, *P* < 0.001). With the SNP active, peak ||GRF|| increased on the first recovery step for both LL1 (5.8 ± 2.7% BW, *P* = 0.033) and LL3 (11.5 ± 2.3% BW, *P* < 0.001) (Fig. 4a, Table S5).

Conversely, prosthetic side perturbations resulted in decreased peak ||GRF|| for LL1 and LL3, evidenced by the significant interaction between the SNP and recovery steps (LL1: F(3, 348) = 5.1, *P* = 0.003; LL3: F(3, 231) = 3.9, *P* = 0.024). Post-hoc analysis revealed decreases in peak ||GRF|| associated with the SNP during the second recovery step for LL1 (6.2 ± 1.7% BW, *P* < 0.001) (Fig. 4b, Table S6) and the third recovery step for LL3 (6.9 ± 2.0% BW, *P* < 0.001) (Table S6).

LL2 exhibited significant effects from recovery steps for both intact (Fig. 4a, F(3, 339) = 925.3, *P* < 0.001) and prosthetic (Fig. 4b, F(3, 288) = 614.8, *P* < 0.001) side perturbations, indicating a recovery response to perturbations to either side. However, no significant effects were observed from SNP activation (intact: F(1, 339) < 0.001, *P* = 0.980; prosthetic: F(1, 288) = 0.4, *P* = 0.548) or its interaction with recovery steps (intact: F(3, 339) = 0.09, *P* = 0.914; prosthetic: F(3, 288) = 0.2, *P* = 0.809).

Comparison of recovery between intact and prosthetic side perturbations revealed SNP-associated changes in the difference between recovery from perturbations to each side for two participants. Significant differences were observed for LL1 (T(119.3) = -5.5, *P* < 0.001) and LL3 (T(151.7) = -11.6, *P* < 0.001) (Fig. 4c). In contrast, LL2 did not experience significant changes (T(159.7) = -1.0, *P* = 0.325). The changes observed with LL1 and LL2 indicated a trend where prosthetic side recovery approached that of intact side recovery when the SNP was active for LL1 and LL3, similar to the changes observed with trunk kinematics.

Interestingly, changes in peak ||GRF|| were accompanied by corresponding instances of LL3 grabbing the treadmill handrails during recovery. For prosthetic side perturbations, LL3’s handrail use decreased from 10 instances with SNP inactive to zero with SNP active. Similarly, for the intact side perturbations, LL3’s handrail use decreased from one instance (SNP inactive) to zero (SNP active). LL1 and LL2 did not use the handrails under any conditions.

In summary, LL1 and LL3 demonstrated increased peak ||GRF|| on the prosthetic side during the first recovery step following intact side perturbations but decreased peak ||GRF|| following prosthetic side perturbations. In addition, the asymmetry in peak ||GRF|| between intact and prosthetic side perturbation responses reduced for LL1 and LL3. These GRF changes were associated with reduced reliance on handrail support for LL3, suggesting improved stability with SNP activation.

## Discussion

In this study, we evaluated the effect of restored plantar sensation from a SNP on the sensorimotor control of individuals with LLL while recovering from treadmill-induced stumbles. Sensorimotor control involves the integration of various sources of sensory inputs resulting in the modulation of motor outputs to maintain smooth and stable gait^26^. Individuals with LLL lack plantar sensation in their missing foot and must rely on limited feedback from the interface between their residual limb and prosthesis socket, so the body may shift the sensory weighting in sensorimotor control away from the residual limb toward more reliable sources of sensory information^27^. When a trip occurs, plantar sensation provides information about how the body is interacting with the environment^4^, which can then be utilized to instinctively slow and arrest the forward rotation and recover without falling. Results reported in this study suggest that the plantar sensation restored by the SNP could be integrated into the sensorimotor control of individuals with LLL to enhance their stability during stumble recovery. These improvements may indicate that, by restoring direct feedback about how pressure is moving along the bottom of the prosthetic foot, the SNP increases confidence in stumble recovery and that it has the potential to reduce the risk of falls for individuals with compromised plantar sensation due to LLL.

When plantar sensation was restored with the SNP, participants decreased their trunk rotation during recovery from stumbles. Aligning with other studies of stumble recovery, the treadmill perturbation induced a forward rotation of the trunk, as seen in Fig. 2a, initiating a recovery response analogous to a trip^24,25^. Trunk kinematics have been identified as important determinants for fall risk^21–23^. To avoid falling after a trip, individuals must slow their body’s forward rotation so that their body’s mass does not project anteriorly enough to cause them to lose balance and fall^28^. The observed reduction in trunk angular sway and peak trunk flexion angular velocity with SNP active indicate improved trunk control during recovery from the applied perturbations. Given that the trunk comprises a significant proportion of total body mass, enhanced control of this segment is crucial for maintaining overall body stability. This improved trunk control suggests a greater capacity to manage the body’s center of mass during unexpected disturbances, which is a critical factor in fall prevention^22^. Consequently, this is a strong indicator of an improved ability to avoid falling, potentially reducing the risk of falls in day-to-day life^21–23^. Therefore, the SNP may contribute to increasing the safety and confidence of prosthesis users and ultimately impact overall activity level and community participation.

This improved trunk control could be attributed to the restored feedback about foot-floor interactions, which provided live and accurate information on how body weight shifted across the foot. When plantar feedback is compromised, the body compensates by increasing its trunk sway to amplify vestibular input^27^. Prior work with the SNP has shown that the converse is also true. When visual and vestibular inputs were compromised, restoring plantar sensation to individuals with LLL allowed the body to shift its sensory weighting toward the restored plantar sensation, ultimately decreasing body sway and improving standing balance^14,18^. Utilization of the SNP appears to enable the central nervous system to redistribute its reliance on sensory information for controlling balance from predominantly prioritizing vestibular input to increasing the sensory weighting of plantar feedback. The changes in trunk kinematics observed with the SNP in this study underscore the significant impact of integrating sensory information from the lower limb into the sensorimotor control of dynamic stability. By altering the contribution of different sensory systems involved in balance, the SNP not only enhances the ability of individuals with LLL to recover from stumbles but also demonstrates the role plantar sensation plays in affecting whole-body dynamics beyond just the amputated leg.

Individuals with LLL must adopt different strategies to recover from perturbations depending on whether they use their intact or prosthetic foot to regain initial stability. Successful recoveries from stumbles require regaining stability as soon as possible, as inadequate initial stability can lead to further imbalance and ultimately result in a fall^5,7^. These recovery strategies differ based on whether individuals can rely on sensation from their intact foot or on the limited feedback from residual-socket interactions for regaining initial stability^9,25^. In our study, we observed the most significant effects of the SNP during the first recovery step, with distinct benefits depending on whether participants relied on their intact or prosthetic foot for this step. These side-specific improvements positively contributed to their stumble recovery for perturbations on either side. Specifically, greater improvements in trunk control were observed in LL1 and LL3 when the SNP was active during the prosthetic side’s first recovery step following an intact side perturbation compared to a prosthetic side perturbation. Without the SNP, both participants exhibited greater trunk flexion when relying on their prosthetic side for their first recovery step. However, when plantar sensation from the prosthetic foot was restored, participants could directly utilize this feedback about their body weight shifting to reduce trunk rotation. This resulted in significant decreases in trunk rotation when recovering on their prosthetic foot, approaching the level of control observed when using their intact foot. These findings demonstrate that the SNP improves the immediate stability in successful stumble recovery through directly restoring crucial sensory information, particularly when the prosthetic limb is used for the first recovery step.

Interestingly, we also observed improvements in trunk control with the SNP even when the prosthetic foot was off the ground and not directly informing the CNS about foot-floor interactions that could influence the immediate recovery maneuver. Previous work from our lab has shown that restoring plantar sensation with the SNP enhances gait adaptations and perception of limb movement, even as the intact limb is in stance and the prosthesis is in swing^16^. It may be that a more accurate perception of prosthetic limb movement can allow participants to better anticipate where their swinging prosthetic foot will contact the ground. This implies that the SNP may be contributing to a more accurate internal representation of their body movement. As such, they may be able to implement better control of their trunk to minimize its rotation before weight acceptance with the prosthetic foot. Alternatively, walking with the SNP may instill a greater sense of trust in the prosthesis due to the ability to sense plantar loading. This may lead to better integration and embodiment of the device and hence improve confidence to sufficiently control whole body movement through coordinated activity of the lower extremity muscles.

After gait perturbations, stepping strategies shift the body’s base of support and redirect GRFs to decelerate and arrest forward rotation. We hypothesized that participants would exhibit responses in peak ||GRF|| corresponding to responses in trunk rotation. However, our observations revealed distinct improvements in the GRFs depending on whether the intact or the prosthetic side was perturbed. Results from prosthetic side perturbations aligned with our hypothesis that the SNP decreased the peak ||GRF||, as observed in the later second and third recovery steps for LL2 and LL3, respectively. The SNP decreased the amount of force applied through the prosthesis to arrest the forward rotation. Restoring plantar cutaneous sensory feedback may allow individuals to rely less on excessive limb loading since they receive accurate sensory cues from their prosthetic foot. In non-amputee populations, enhancing plantar sensation has similarly been associated with reduced rates of limb loading following postural perturbations^7^. Plantar sensation provides critical information about foot placement, heel strike timing, and body-weight transfer during stance, enabling more precise modulation of braking forces needed to regain stability. When the prosthetic foot is perturbed, the SNP supplies the user with information regarding limb interactions with the ground. With this enhanced sensory context, the user can more effectively calibrate their corrective response, reducing peak ||GRFs|| during subsequent recovery steps to regain stability after the perturbation.

For intact side perturbations, the SNP increased the peak ||GRF|| under the prosthetic foot on the first recovery step, contrary to our original hypothesis. When comparing responses between intact side and prosthetic side perturbations with the SNP inactive, the peak ||GRF|| under the intact foot on the first recovery step after a prosthetic side perturbation was already higher than the peak ||GRF|| on the first recovery step made by the prosthetic foot after an intact side perturbation. This suggests that individuals with LLL may lack confidence in their prosthesis while recovering from intact side perturbations, attempting to minimize the weight placed on it in the absence of reliable sensory feedback. Notably, with the SNP active, not only did peak ||GRF|| on the prosthetic limb increase after an intact side perturbation, but this increase approached the peak ||GRF|| of the intact limb after a prosthetic side perturbation. This suggests that the SNP enhanced the symmetry of the loading responses during recovery with the prosthetic foot toward those of recovery with the intact foot, which may contribute to greater stability. This behavior suggests that the SNP enables users to rely on their prosthetic feet in a manner similar to their intact feet. Restoring sensation with the SNP may be allowing for the sensorimotor control system to reweight its summation of sensory signals and increase its reliance on plantar sensation from the prosthetic foot, similar to observations during challenges to standing balance in a sensory organization test^14^. Results of peak ||GRF|| showed that the SNP helped users shift their reliance away from their intact foot (resulting in a decrease in the ||GRF||) and onto their prosthetic foot (resulting in an increase in the ||GRF||).

A more symmetric recovery response implies that participants were more confident in their prosthetic limbs after experiencing destabilizing perturbations. This is not only evident in peak ||GRF||, but also supported by the greater symmetry in trunk control. Although trunk sway and trunk flexion angular velocity decreased for both intact and prosthetic side perturbations, analysis of the first recovery step revealed that, with the SNP, responses using the prosthetic foot for stance more closely resembled those using the intact foot for stance. Shirota et al*.* found that individuals with transfemoral amputations adopted different recovery strategies than able-bodied individuals when their limbs were physically obstructed^9^. This may have been to place their intact foot on the floor as soon as possible because of the inherent confidence in utilizing intact sensation from that foot for recovery. While the balance confidence and fear of falling of participants in our study were not directly assessed, our results indicated that restoring sensation with the SNP allowed individuals to rely on their prosthetic foot more when recovering from perturbations than with the SNP inactive. This enhanced confidence is reflected in both the decreased trunk rotation and more symmetric GRF response on the prosthetic foot with the SNP active.

Further evidence of increased confidence and reliance on the prosthetic foot is evident in the number of times the treadmill handrails were grabbed. LL1 and LL2 (participants with transtibial limb loss) did not reach for the handrails at all during our experiments, whereas LL3 (the participant with knee disarticulation) grabbed the handrails 10 times during prosthetic side perturbations and once during intact side perturbations when the SNP was inactive. When the SNP was active, LL3 did not grab the handrails at all. This indicates that LL3 may have lacked confidence in their ability to successfully recover from the perturbations without the SNP. Because they required both a prosthetic knee and ankle, joint orientation feedback from the knee which was still intact for LL1 and LL2 was unavailable to LL3. This may have contributed to LL3 having less trust in recovering from perturbations without sensory feedback than either LL1 or LL2. This confidence may be even lower for perturbations initiated on the prosthesis side because of the inability to accurately detect and rapidly respond to the start of the perturbations on the stance limb due to the lack of feedback from both the knee and ankle joints. The SNP enhanced their confidence in their prosthesis such that they no longer needed to grab the handrails to restore their balance.

Psychological factors, such as fear of falling and embodiment, can affect an individual’s ability to recover from stumbles. Higher levels of fear of falling have been linked to reduced gait stability and balance^29^. As described above, by enabling users to rely more confidently on their prosthetic limb, the SNP may help mitigate these concerns. Prosthesis embodiment, in particular, is crucial for establishing a robust sense of prosthetic foot position in space for the prosthesis user^3^. Previous research has shown that providing sensory feedback to individuals with lower limb amputation can reduce the perceived weight of their prosthesis^30^ and improve the perception of limb speed^16^, both recognized indicators of improved prosthesis embodiment. Although embodiment was not directly measured in this study, our results suggest that the plantar sensory feedback facilitated a more natural integration of the prosthesis into the user’s sensorimotor control. Notably, the reduced trunk rotation observed even when the prosthetic limb was in swing (and not directly providing restored plantar sensory feedback) points to the development of a more accurate internal body representation of the prosthetic limb. This improved body schema likely enhances motor planning and overall embodiment of the prosthesis^31^.

One participant (LL2) did not experience the same functional effects of the SNP on their recovery biomechanics. After an intact side perturbation, LL2 produced small but significant increases in trunk sway and peak trunk flexion angular velocity with the SNP active during the first recovery step with the prosthetic side but did not exhibit any significant effects from the SNP on their peak ||GRF||. Although the increase in trunk angular sway and peak trunk flexion angular velocity when the SNP was active was opposite the decrease seen with LL1 and LL3, the magnitudes of these increases were much smaller in comparison. Furthermore, these increases were not associated with changes in peak ||GRFs||. Therefore, we concluded that the SNP did not provide LL2 with significant functional changes to their stumble recovery strategy. This lack of effect may be attributed to inter-individual differences in the magnitude of perturbations required to elicit a significant recovery response. For LL2, who routinely walks long distances without incidents of falls or trips, a higher magnitude of perturbation might have been necessary to observe SNP-related effects. However, the treadmill utilized for all three participants in this study was limited to a maximum acceleration of 3 m/s^2^ and may have been unable to provide a challenging enough perturbation for the SNP to have observable effects on the recovery response of LL2. Despite having a similar lower limb amputation level, LL1 also has unilateral upper limb loss. Whole body coordination, including the upper limbs, has been shown to be important in for recovering from trips and avoiding falls^32^. This may have impaired LL1’s ability to recover from trips, making the present perturbations sufficiently strong for the SNP to have an effect on their recovery. LL3 has a knee disarticulation, yet still exhibited similar improvements in recovery with the SNP active as LL1. The lack of sensory input from the missing knee may have therefore made LL3 as vulnerable to stumbling with the available treadmill belt speed perturbations as LL1. LL2’s trunk kinematics when the SNP was inactive indicated they already had smaller trunk rotation than LL1 and LL3, reinforcing that LL2 already had greater overall stability when recovering from perturbations. Consequently, both LL1 and LL3 may have been more challenged than LL2 with the magnitude of the perturbations delivered in this study.

The SNP technology restores information about the magnitude and distribution of pressure along the plantar surface of the prosthetic foot. While it does not directly restore proprioceptive information (i.e. joint movement and position) which can be critical for sensing body movement, plantar pressure information alone can help the body infer about how weight shifts under the foot during walking. Importantly, it provides accurate information about the timing and location of heel contact and toe-off. This enhanced spatial awareness may explain the improved stumble recovery with the SNP. Restoring accurate foot placement information could contribute to a more accurate internal model of the body, allowing users to modulate their motor outputs more effectively for recovery. This effect is particularly evident in the decreased trunk sway and trunk flexion velocity. Further research is needed to explore this concept, and the SNP can be a valuable tool for investigating interactions of internal models, reflex circuitry, and sensorimotor integration, among other factors contributing to gait stability, stumble recovery, and fall prevention.

For individuals with transtibial amputations, consistent trip-specific training over a period of 2 weeks has also been shown to reduce the peak trunk flexion angular velocity^25^. Our participants achieved similar benefits of reduced trunk flexion angular velocities through restoring sensation related to their missing feet with the SNP, without requiring such a training program. While our reported improvements in trunk flexion angular velocities are smaller than those reported after trip-specific training in Kaufman et al.^25^, it is important to note the difference in perturbation magnitudes between studies, preventing direct comparison of the effects reported from other studies with the effects of the SNP that we observed. Nevertheless, we report significant improvements in trunk control when the SNP was active in two out of three participants. Future work should be directed toward studying the interactions between treadmill belt speed and acceleration on recovery responses with and without sensory feedback via neural stimulation.

Trip-specific training protocols, which include repeated exposures to treadmill perturbations, have been shown to induce long-term motor learning. Such training improves recovery from stumbles and reduces the instances of falls, and these improvements can persist for at least six months^25^. Although this study focused on the short-term improvements in stumble recovery without implementing a dedicated trip-specific training regimen, previous qualitative work from our group suggests that long-term use of the SNP may result in sensorimotor learning in individuals with lower limb amputation^33^. Building on these findings, it would be valuable for future research to investigate how integrating the SNP into trip-specific training might sustain or enhance these benefits over time.

The treadmill perturbations used in our experiments have been shown to evoke trip-like forward rotations of the body and evoke reflexes in the calf muscles and the tibialis anterior^24,34^. However, unlike many real-world trips, they did not physically obstruct the limbs of our participants. Physical obstructions can generate noxious cutaneous inputs from the toes or an unexpected quadriceps muscle stretch, potentially triggering spinal reflex mechanisms that were not elicited in this study. Future studies could examine the effect of the SNP on short-latency cutaneous reflex mechanisms. Previous work in our lab has shown that the electrically restored sensation from the SNP does not interfere with reflex mechanisms during postures that mimic gait^17^. Although characterizing reflexive responses during stumble recovery was outside the scope of our current study, these reports strongly suggest that the SNP is integrated into the sensorimotor control system and may be incorporated into the body’s reflex circuitry that maintains dynamic stability.

Prosthetic alignment and prosthesis type have also been shown to impact gait and balance. For example, changes in the alignment angle for a prosthetic ankle or knee can alter the loading pattern on the prosthetic limb^35^, potentially contributing to between-individual differences in stumble recovery. In our study, participants utilized their clinically prescribed prostheses which they had worn for at least five years prior to the start of this study. While these pre-existing prosthesis characteristics may have affected responses between individuals, it is important to note that the prosthesis itself remained unchanged across SNP conditions within each participant. Therefore, the changes we observed between SNP conditions can be attributed to the effects of the SNP rather than variability in prostheses.

This study included three individuals with LLL exhibiting varying amputation levels, prostheses, and experience with the SNP. Improvements in stumble recovery were observed in two of the three participants, suggesting that the SNP has the potential to benefit a large proportion of lower limb prosthesis users regardless of prosthesis type and alignment, and may be particularly valuable to those with transfemoral limb loss. However, validation of our results in a larger sample of lower limb prosthesis users is necessary. Furthermore, the benefits of improved plantar sensation may extend to other populations with impaired foot sensation, such as the elderly and individuals with peripheral neuropathy. This assertion has been supported by previous reports showing that improving plantar perception in the elderly can enhance their stability and their recovery from forward falls^7,8^. The impact of the SNP on balance, gait, and stumble recovery of individuals with intact lower limbs but documented sensory impairments remains to be determined and warrants further investigation.

In conclusion, restored plantar somatosensory feedback with the SNP enhances stumble recovery performance in lower limb prosthesis users. This study used established methods of evoking stumble recovery responses on a treadmill, and the findings indicate that the SNP can be integrated into the sensorimotor control scheme to enhance the reliance on and confidence with using the prosthesis for stumble recovery. Ultimately, this resulted in more stable recovery in response to the perturbations as evidenced by improved trunk control and more symmetrical GRF responses. By providing direct and accurate feedback about how the body weight is shifting along an individual’s foot, the SNP may reduce the risk of falls for individuals with LLL and provide lower limb prosthesis users with the confidence to safely navigate unfamiliar terrain in their communities.

## Methods

### Participants

Three male participants with unilateral lower limb amputations were recruited to this study. LL1 (62 years old, height: 1.68 m, mass: 71.7 kg) and LL2 (62 years old, height: 1.85 m, mass: 85.1 kg) had transtibial amputations, and LL3 (52 years old, height: 1.98 m, mass: 108.9 kg) had a knee disarticulation amputation. All participants wore their clinically prescribed prothesis during the experiments. LL1 routinely used a powered ankle prosthesis (Ottobock Empower) that provides push-off assistance calibrated for predictable, steady-state gait conditions. We instructed LL1 to deactivate the powered features because the timing of this motorized assistance may be misaligned with unexpected treadmill perturbations, potentially disrupting recovery strategies or putting the user at unnecessary risk. Consequently, LL1’s device functioned as a passive prosthesis throughout the experiment, similar to the devices used by the other participants (LL2: Össur Pro-Flex XC Foot; LL3: Össur RHEO Knee). Although we did not directly compare participants with one another, maintaining passive device functionality helped ensure that any observed improvements could be more consistently attributed to SNP intervention, rather than to variability in prosthetic device features. Participants did not have any medical history of neuropathy or peripheral vascular disease. LL1, LL2, and LL3 have been living with limb loss for 16, 15, and 5 years, respectively, prior to the start of the study. All participants were K3 level ambulators. All experimental procedures were approved by the Louis Stokes Cleveland Veterans Affairs Medical Center Institutional Review Board and conducted under an Investigation Device Exemption from the United States Food and Drug Administration (IDE G110043), which governed use of the neural stimulation technology. Participants gave their written informed consent prior to any research-related activities, following relevant human subject protection guidelines and regulations.

### Sensory neuroprosthesis (SNP)

The SNP consists of implanted electrodes, an instrumented prosthesis, and an external stimulation controller. Each participant received 16-contact composite flat interface nerve cuff electrodes (C-FINEs) surgically installed around their remaining sciatic and/or tibial nerves in their residual limb above the knee (Fig. 1a). The C-FINEs were connected to percutaneous leads exiting the body in the upper thigh for LL1 and LL2 with transtibial limb loss, or lower abdomen for LL3 with the knee disarticulation. Details of the neural interface technology and the surgical procedure are described elsewhere^13^. A custom-made and optically isolated external stimulator delivered current-controlled asymmetric charge-balanced cathodic-first waveforms to selected contacts within the C-FINEs with pulse amplitudes ranging between 0.8 and 2 mA and pulse widths ranging between 0 to 255 μs. The inter-pulse interval was maintained at 50 ms, consistent with prior reports. To elicit plantar sensations from the missing foot, electrical stimulation was delivered to contacts in the electrodes around the tibial nerve in LL1 and the sciatic nerve in LL2 and LL3. The contacts and the stimulation parameters were chosen based on prior thresholding and mapping experiments, which have been previously reported^13–15^. Electrical stimulation parameters were systematically varied across electrode contacts, and participants were instructed to describe the elicited sensations in their own words and report the intensity of the sensations based on their own self-selected scale^13^. By identifying the electrode contacts and parameters that consistently evoked sensations in the missing heel, midfoot, and metatarsal regions, we ensured that the stimulation delivered during the present study would reliably produce the intended sensory perceptions. LL1, LL2, and LL3 had received the implanted components for 6 years, 5 years, and 5 months prior to the start of the study, respectively.

A force-sensing insole (High Dynamic Force Sensing Resistor Insole, IEE, Bissen, Luxembourg) consisting of eight discrete force sensing resistors (FSRs) was attached to the bottom of the prosthetic foot, and data from the insole were collected and transmitted wirelessly to the external stimulation controller via a custom-made lightweight electronic module that attached to the prosthesis pylon (Fig. 1a). These eight discrete FSRs were divided into three distinct regions: heel (two FSRs), midfoot (one FSR), and metatarsal (five FSRs). Data from each set of sensors were collected continuously and averaged within their respective region (Fig. 1b). This setup allowed us to align the pressure signals from the prosthetic foot with the restored plantar sensations reported by the participants, as determined through the thresholding and mapping experiments. The external stimulation controller, as described above, delivered electric pulses to C-FINE contacts based on real-time foot-floor interactions. Elicited sensations resulting from the neural stimulation correlated in location and intensity with the pressure detected within specific plantar regions of interest: the heel, midfoot, and metatarsal regions of the foot as illustrated for each participant in Fig. 1b. This ultimately allowed the participants to feel how their body weight shifted along the prosthetic foot in the anterior–posterior direction. Details of this complete SNP system configuration have been previously reported^16^. All participants had ample experience standing and walking with the SNP overground and on a treadmill.

### Experiment setup and data collection

All walking and perturbation experiments were performed on a split-belt instrumented treadmill (R-Mill, ForceLink, Netherlands) in a virtual reality environment (V-Gait, Motek Medical, Netherlands). The treadmill’s belts were always driven at the same speed during steady state, unperturbed walking for each participant, and they had a maximum acceleration of 3 m/s^2^ for applied perturbations. The treadmill speed was controlled using the D-Flow software (version 3.34.3, Motek Medical, Amsterdam, Netherlands). Visual flow was provided by a virtual reality projection that simulated walking down an outdoor path. Participants wore a safety harness while walking on the treadmill. Force data were recorded from two force plates embedded in the treadmill at a 1000 Hz sampling frequency. Kinematic data were recorded using a full-body Vicon Plug-In Gait marker set, which included 44 reflective markers. Marker trajectories were captured with a 16-camera motion capture system at a 100 Hz sampling frequency (Vicon, Oxford, UK).

### Experiment design and perturbation mechanism

Prior to perturbation trials, each participant’s preferred walking speed on the treadmill was determined. The treadmill belt speed was initially brought to either 0.5 m/s or 1.5 m/s. Participants could adjust the speed by saying “up” to increase or “down” to decrease the speed, with changes made in randomly varying increments (smallest increment being 0.05 m/s) until they could comfortably walk for 30 s. This process was repeated 10 times, five times starting at each initial speed setting (0.5 m/s or 1.5 m/s), to determine average preferred walking speed from the final speeds across all trials.

During sessions, participants were advised to maintain balance without grabbing the handrails, although they could use them if necessary to regain balance. To accustom the participants to treadmill walking, they walked at their preferred walking speed for at least two minutes without any perturbations. Perturbations were then introduced randomly to the left and right sides during a maximum of 10 additional minutes of walking, spaced at least 10 strides apart to allow a return to steady-state walking between perturbations. On average, 12 perturbations were delivered to each side during a single block of treadmill walking.

The perturbation mechanism involved both treadmill belts accelerating at the maximum allowable 3 m/s^2^ and then decelerating at the same rate to return to the participant’s preferred walking speed, each phase lasting half the perturbation duration. An example of the perturbation can be seen in Fig. 2a and b^24,36^. These perturbations were programmed to trigger in early stance when the vertical GRF on the perturbed side exceeded 100 N, ensuring that participants were in a consistent phase of gait before each perturbation. Previous studies have successfully employed perturbation parameters of comparable magnitude to induce forward rotations of the trunk, mirroring the biomechanical conditions associated with an actual stumble^24,36^. The perturbation duration was selected to elicit a recovery response without forcing the participant to use handrails for support and was selected to allow the treadmill belts to return to the preferred walking speed by the time the participant completed a single stride with the perturbed foot. The duration of each perturbation was 800 ms for LL1 and LL2, and 600 ms for LL3 (Fig. 2c). Preliminary testing revealed that the original 800 ms perturbation duration was too intense for LL3 because they frequently relied on the handrails during recovery. To ensure that LL3 could safely recover without external support and still produce a meaningful stumble response, we reduced the perturbation duration to 600 ms. Although this adjustment represented application of a different challenge than the condition used for LL1 and LL2, the perturbation duration remained consistent across SNP inactive and active trials for LL3, allowing for valid within-participant comparisons.

### Data analysis

Following data collection, reflective markers affixed to participants were digitally labeled in Vicon Nexus software (version 2.14.0, Vicon, Oxford, UK) to ensure precise tracking of anatomical landmarks. Occasional gaps in marker data were interpolated using the same software. A participant-specific rigid body model was constructed in Visual3D (HAS-Motion, Ontario, Canada) for detailed motion analysis. Marker data and force plate data were low pass filtered at a cutoff frequency of 6 Hz and 10 Hz, respectively, with 4^th^ order low pass Butterworth filters. A 3^rd^ order median filter was applied to the data to remove impulse noise from the signals. Further analyses to compute and extract outcome measures were performed in MATLAB (R2020b, MathWorks, Natick, MA).

Heel contact and toe off events were identified by an established algorithm applied to the data from each foot’s heel and toe markers, respectively^37^. Force and moment data collected during trials were normalized relative to each participant’s body weight, calculated from the average magnitude of the sum of the forces on the two force plates embedded in the treadmill while participants stood still in a T-pose. These static trials were also used to calibrate the rigid body models in the motion analysis.

The stages of recovery were broken down into the perturbation, first recovery, second recovery, and third recovery steps (intact side perturbation: Fig. 2a, prosthetic side perturbation: Fig. 2b). The perturbation step was defined as the step taken by the limb while the treadmill belt accelerated and decelerated. The first recovery step began with the first heel strike of the non-perturbed (i.e. contralateral) side following the perturbation. The second and third recovery steps followed sequentially, involving the perturbed side and then non-perturbed side, respectively. Thus, in examining the recovery dynamics following the perturbation, we analyzed the sequence from the perturbation through the first to the third recovery steps. Preliminary analysis showed that most biomechanical parameters returned to baseline by the end of the third recovery step. Therefore, we focused on steps from the initial perturbation up to the third recovery step, as ineffective balance restoration in initial steps often necessitates greater compensatory adjustments in subsequent steps^5^.

Trunk angular sway, trunk flexion angular velocity, and peak ||GRF|| were analyzed. The trunk angle was quantified as the angle between the torso and the global vertical axis, consistent with established measurements of trunk kinematics during stumble recovery^24,25^, and trunk angular velocity, representing the rate of change of trunk angle, was calculated as its time derivative. The trunk angular sway for each step was calculated as the range of trunk angle observed during the step. Peak trunk flexion angular velocity was determined by identifying the trunk angle maxima in the trunk flexion direction within each step. The ||GRF|| on each side was calculated as the magnitude of the GRF vector. The peak ||GRF|| for a step was defined as the value of the first peak of the ||GRF|| on each side’s associated step.

Data from the first two perturbations to each side of every block of walking were excluded to remove any initial learning effect related to novel application of the perturbations. Data were included in the analysis only if the perturbation was initiated within the first 20% of the gait cycle and if the participant did not grab the handrail, as indicated by hand marker data. For trunk kinematics, only recovery datasets with complete marker data were used. Any datasets with more than 5% of the marker data missing were excluded to maintain the integrity of analysis. Data for ||GRF|| were included only if the participant did not cross the treadmill’s mid-line which would result in inaccurate force measurements. This was ensured by verifying that the vertical GRF on each force plate remained below 20 N (i.e. a force plate detected the leg was in swing) for at least 15% of the gait cycle.

Data were also excluded if a perturbation resulted in loss of balance where the participant did not feel confident recovering on their own and grabbed the handrails. This was detected from the finger marker speed by finding the magnitudes of the finger marker velocities from the motion capture data. A participant was considered to have grabbed the handrail if the finger marker speed was less than 5 cm/s for at least 50 ms.

### Statistical analysis

Separate statistical analyses were performed on intact and prosthetic side perturbations to accurately reflect biomechanical distinctions. All analysis was run in MATLAB (R2020b, Mathworks, Nattick, MA). A repeated measures analysis of variance was used with recovery step (perturbation, first recovery, second recovery, third recovery) as the repeated measures factor and SNP condition (active or inactive) as the between-group factor. Data normality was assessed using Q-Q plots, and sphericity was evaluated using Mauchly’s test. Any violations in the sphericity assumption were corrected with the Greenhouse–Geisser correction. Differences within recovery steps due to SNP condition were further explored using a multiple comparison post-hoc analysis with Bonferroni correction. Since the SNP had the most effect during the first recovery step, we also performed a Welch’s t-test to determine if differences in this step between the intact and prosthetic side perturbations were affected by the SNP for each participant. The number of times each participant grabbed the handrails over the duration of the experiment was counted for each perturbation side and SNP condition.

## Electronic supplementary material

Below is the link to the electronic supplementary material.

Supplementary Material 1

## Data Availability

Data collected for this study and the corresponding code used to analyze the data can be found at 10.5281/zenodo.13879472.
