# Supplementary material for: A sensory neuroprosthesis enhances recovery from treadmill-induced stumbles for individuals with lower limb loss
Source: Sci Rep. 2025 Jan 11;15:1732. doi: 10.1038/s41598-025-85788-4 (PMC11724839; doi:10.1038/s41598-025-85788-4)
Supplement: Supplementary file 1 — Supplementary Material 1 [file 41598_2025_85788_MOESM1_ESM.docx]

For all the supplementary tables below, * denotes a statistically significant difference (P < 0.05) between the SNP inactive and active conditions across recovery steps, as determined by the post-hoc multiple comparisons tests.

**Table S1.** Trunk angular sway (°) after intact side perturbations (mean ± standard deviation).

|  | | **LL1** | | **LL2** | | **LL3** | |
| --- | --- | --- | --- | --- | --- | --- | --- |
| **SNP** | | Inactive | Active | Inactive | Active | Inactive | Active |
| **Step** | *n* | 58 | 59 | 62 | 64 | 48 | 53 |
| Perturbation | | 3.4±0.9 | 3.3±0.8 | 3.3±1.1 | 3.1±0.8 | 6.1±1.4 | 6.1±1.3 |
| 1^st^ Recovery | | 9.8±1.9* | 8.6±1.9* | 5.2±1.4* | 5.7±1.2* | 14.7±3.5* | 9.3±2.0* |
| 2^nd^ Recovery | | 6.1±1.9 | 5.8±1.7 | 2.7±0.8 | 2.5±0.8 | 9.5±1.7* | 7.3±1.5* |
| 3^rd^ Recovery | | 4.2±1.1 | 3.9±1.1 | 3.9±1.0 | 3.9±0.9 | 7.1±3.4* | 4.0±0.8* |

**Table S2.** Peak trunk flexion angular velocity (°/s) after intact side perturbations

|  | | **LL1** | | **LL2** | | **LL3** | |
| --- | --- | --- | --- | --- | --- | --- | --- |
| **SNP** | | Inactive | Active | Inactive | Active | Inactive | Active |
| **Step** | *n* | 58 | 59 | 62 | 64 | 48 | 53 |
| Perturbation | | 16.1±6.4 | 18.0±5.1 | 15.4±4.9 | 14.7±4.0 | 24.9±6.7 | 23.8±6.1 |
| 1^st^ Recovery | | 91.3±11.4* | 80.8±11.1* | 30.1±6.2* | 32.6±6.7* | 66.8±17.4* | 39.9±13.4* |
| 2^nd^ Recovery | | 3.2±6.1 | 4.5±6.3 | 11.1±5.5 | 11.5±5.9 | 38.9±11.1* | 27.3±7.7* |
| 3^rd^ Recovery | | 13.9±5.9 | 14.5±4.7 | 9.6±4.0 | 8.5±3.5 | 21.1±9.7 | 19.0±6.4 |

**Table S3.** Trunk angular sway (°) after prosthetic side perturbations

|  | | **LL1** | | **LL2** | | **LL3** | |
| --- | --- | --- | --- | --- | --- | --- | --- |
| **SNP** | | Inactive | Active | Inactive | Active | Inactive | Active |
| **Step** | *n* | 62 | 62 | 66 | 65 | 44 | 52 |
| Perturbation | | 2.4±0.7 | 2.4±0.8 | 3.3±0.8 | 3.2±0.8 | 5.3±1.8 | 4.7±1.7 |
| 1^st^ Recovery | | 11.1±2.1* | 9.8±2.2* | 9.2±1.7 | 9.5±1.8 | 12.0±3.7* | 9.5±2.7* |
| 2^nd^ Recovery | | 7.0±1.8 | 6.6±1.7 | 4.6±1.4 | 4.5±1.3 | 7.5±1.4* | 6.2±1.4* |
| 3^rd^ Recovery | | 4.2±1.3 | 4.1±1.4 | 2.6±0.9 | 2.7±0.7 | 6.4±1.4 | 6.1±1.3 |

**Table S4.** Peak trunk flexion angular velocity (°/s) after prosthetic side perturbations

|  | | **LL1** | | **LL2** | | **LL3** | |
| --- | --- | --- | --- | --- | --- | --- | --- |
| **SNP** | | Inactive | Active | Inactive | Active | Inactive | Active |
| **Step** | *n* | 62 | 62 | 66 | 65 | 44 | 52 |
| Perturbation | | 19.3±3.9* | 16.0±5.6* | 10.1±6.9 | 9.8±7.0 | 26.3±10.0 | 24.5±10.2 |
| 1^st^ Recovery | | 52.5±9.9 | 49.2±10.4 | 50.6±9.8 | 51.9±9.2 | 57.8±16.0* | 47.5±11.8* |
| 2^nd^ Recovery | | 19.8±9.6* | 13.8±8.5* | 5.9±4.8 | 6.7±4.3 | 25.7±8.7 | 22.9±6.8 |
| 3^rd^ Recovery | | 17.2±7.6 | 15.2±9.0 | 12.8±4.2 | 11.6±5.0 | 26.4±8.4* | 21.7±6.5* |

**Table S5.** Peak ground reaction force magnitude (% body weight) after intact side perturbations

|  | | **LL1** | | **LL2** | | **LL3** | |
| --- | --- | --- | --- | --- | --- | --- | --- |
| **SNP** | | Inactive | Active | Inactive | Active | Inactive | Active |
| **Step** | *n* | 54 | 68 | 57 | 58 | 44 | 50 |
| Perturbation | | 117.0±2.8 | 116.0±5.7 | 105.7±4.1 | 105.7±4.5 | 99.4±5.8 | 99.8±6.3 |
| 1^st^ Recovery | | 168.2±17.8* | 174.0±12.0* | 140.0±7.7 | 140.1±7.6 | 123.2±11.6* | 134.8±11.0* |
| 2^nd^ Recovery | | 136.4±10.4 | 136.4±8.1 | 116.2±7.3 | 116.5±7.5 | 117.2±8.2 | 115.3±6.0 |
| 3^rd^ Recovery | | 109.8±6.7 | 109.5±7.1 | 104.0±3.7 | 103.6±3.1 | 100.5±12.1 | 98.2±7.7 |

**Table S6.** Peak ground reaction force magnitude (% body weight) after prosthetic side perturbations

|  | | **LL1** | | **LL2** | | **LL3** | |
| --- | --- | --- | --- | --- | --- | --- | --- |
| **SNP** | | Inactive | Active | Inactive | Active | Inactive | Active |
| **Step** | *n* | 57 | 61 | 49 | 49 | 37 | 42 |
| Perturbation | | 107.8±4.8 | 108.0±4.6 | 104.4±3.0 | 104.3±3.0 | 95.4±4.1 | 95.5±3.8 |
| 1^st^ Recovery | | 178.3±6.0 | 178.6±6.7 | 144.8±12.2 | 144.0±9.2 | 130.3±8.3 | 130.3±6.4 |
| 2^nd^ Recovery | | 133.3±9.1* | 127.1±9.5* | 109.9±6.9 | 110.1±6.9 | 126.0±15.6 | 118.8±17.3 |
| 3^rd^ Recovery | | 120.3±7.3 | 119.5±7.2 | 108.8±7.4 | 107.6±6.2 | 113.0±10.1* | 106.1±7.2* |
